# Design and validation of nurses’ knowledge, attitude and practice questionnaire for discharge planning of stroke patients

**DOI:** 10.3389/fneur.2025.1583169

**Published:** 2025-07-31

**Authors:** Su Fu, Shanshan Liu, Lijuan Duan, Chaofeng Fan, Yan Jiang

**Affiliations:** ^1^Department of Neurosurgery, West China Hospital, Sichuan University, Chengdu, Sichuan, China; ^2^West China School of Nursing, Sichuan University, Chengdu, Sichuan, China

**Keywords:** knowledge-attitude-practice, discharge planning, stroke patients, nurses, validation

## Abstract

**Objective:**

The aim of this study was to design a national and culturally appropriate questionnaire to assess the nurses’ knowledge, attitude and practice (KAP) for discharge planning of stroke patients and to test the reliability and validity of this questionnaire.

**Methods:**

This questionnaire was developed using domestic and international literature and combining the KAP theory. It covered eight aspects of basic knowledge, relevant knowledge, psychological cognition, emotional tendency, intentional behavior, assessment, implementation and evaluation of discharge planning for stroke patients. The item pool was established through Delphi expert inquiry to form a pre-survey questionnaire. The pre-survey was first administered to 60 clinical nurses, then tested on 626 clinical nurses. Finally the questionnaire’s reliability was assessed through retest reliability, split-half reliability, and Cronbach’s *α* coefficient, while the validity of the scale was evaluated through content validity, structural validity, and factor analysis.

**Results:**

The final formal questionnaire was used to assess nurses’ KAP for discharge planning of stroke patients. The questionnaire consisted of 37 items in three dimensions: knowledge (12 items), attitude (10 items) and practice (15 items). The questionnaire had an average expert authority coefficient of 0.88, a consistency coefficient of 0.454, a total internal consistency reliability (Cronbach’s alpha coefficient) of 0.909, a retest reliability of 0.924, and a content validity index of 0.972. The results of this study were calculated on a dimensional basis, resulting in the following standard scores: 84.01 (<85) for the knowledge dimension, 90.61 (>85) for the attitude dimension, and 81.83 (<85) for the practice dimension. The correlation coefficients between the three dimensions and the total questionnaire were 0.635, 0.659, and 0.566, respectively. The Kaiser-Meyer-Olkin (KMO) value was 0.966, and the sphere test value was *p* < 0.0001.

**Conclusion:**

The questionnaire on nurses’ KAP for discharge planning of stroke patients showed good reliability and validity. It is suitable for use by clinical nurses. Nurses in Level-3 hospitals across the country need to improve their knowledge, attitude and practice regarding discharge planning for stroke patients.

## Introduction

Discharge planning is a fundamental component of continuity of care and rehabilitation facilitation. It refers to the process required for the successful transfer of a patient from one healthcare facility to another setting (hospital, nursing home, home) (1), as well as the precise development of a plan and assistance in the appropriate treatment and care of the patient after discharge (2). Discharge planning has been established as a mandatory and indispensable part of the healthcare delivery system in Europe and the United States (3, 4). In China, although discharge planning is not integrated into the national health management system and medical care routines, major public hospitals have been vigorously promoting discharge planning programmes among chronically ill patients in recent years. Discharge planning is the basic guarantee of effective, rational and continuous treatment and care (5). A rational discharge planning programme enables stroke patients to establish and implement a discharge planning programme from the beginning of their admission to hospital, enables discharged patients to receive the necessary long-term treatment services, improves stroke patient adherence, and reduces readmission rates (6). Stroke patients in trial group group who implemented a discharge planning had a lower readmission rate at 6 weeks post-discharge (8.1% in the trial group vs. 16.3% in the control group, *p* = 0.048) (7). Therefore, discharge planning interventions for stroke patients can reduce the length of stay, improve self-care, and increase patients’ satisfaction with care (8).

The implementation of discharge planning involves four processes: assessment, planning, implementation and evaluation (9). Nurses have the closest contact with patients during hospitalisation or after discharge. Discharge planning is a bridge between the hospital and follow-up care, in which nurses play an important role. In foreign countries, the government requires nurses to perform discharge planning services, and stroke discharge planning is performed by advanced practice nurses (10). However, there is no uniform requirement in China. According to the literature (11), nurses are the only professional and technical staff who can take the lead in implementing discharge planning. The implementation of nurse-led discharge planning can reduce the readmission rate and all-cause mortality rate, which has a positive significance in improving patients’ quality of life. The influencing factors for the implementation of discharge planning are mainly the age of nurses, the type of institution, the ratio of doctors and patients, the importance of hospitals, and the attitudes of patients and their families, and that nurses’ knowledge and attitudes towards discharge planning directly affect the effectiveness of discharge planning implementation (12–14). In addition, there are many studies on nurses’ knowledge, attitude, and practice (KAP) regarding discharge planning, and it has been reported (15–17) that only 20% of nurses were able to clearly define the content of discharge planning, 67% of nurses believed that discharge planning should be started on the day of discharge, and 76% of nurses believed that one of their roles was to prepare the patient for discharge from the hospital. It can be seen that the focus of foreign research on nurses in discharge planning has concentrated on nurses’ KAP towards discharge planning, and nurses’ preparation for discharge planning has a direct impact on the outcome of patients’ discharge (18).

However, at present, there is no uniform and systematic perception and management of the specific content and implementation process of discharge planning in China. It is only blindly referenced and tentatively explored (9). Studies on nurses’ KAP towards discharge planning for stroke patients have not been reported in China. Therefore, this study investigated the current status of nurses’ KAP towards discharge planning for stroke patients, developed and tested the reliability and validity of a national and culturally appropriate KAP questionnaire for use by clinical nurses in discharge planning for stroke patients.

## Materials and methods

### Development of the nurses’ KAP questionnaire for discharge planning of stroke patients

#### Establishment of item pool

Pubmed, Medline and Embase databases were searched. The search period was from the creation of the database to 30 November 2021. Search fields included “stroke, apoplexy, cerebrovascular accident, cerebrovascular stroke, brain vascular accident, cerebral stroke, acute. Stroke, cerebral hemorrhage, patient discharge, discharge planning, discharge, patient, discharge of patients, discharge service, discharge preparation service, nurses’ perception, nurses’ attitudes, nurses’ knowledge, knowledge and practice towards discharge planning.” The study team consisted of one stroke ward manager, one stroke medicine specialist, one evidence-based nursing specialist, one clinical nurse on the stroke ward, and one nursing researcher, for a total of five members. All team members were well versed in the Delphi expert consultation method. The team extracted scale items from the three dimensions of nurses’ KAP regarding discharge planning for stroke patients through the literature review, taking into account the national context. The team discussed the development of the initial nurses’ KAP questionnaire about discharge planning for stroke Patients.

#### Delphi expert consultation

Delphi is a method of judgement based on the opinions and values of experts, and the qualification of experts is an important factor that affects the reliability of research results (19). The qualifications of experts were mainly considered in the following aspects: field of expertise, relevant work experience, title, education, and their willingness (20). According to the content of this study, experts with relevant work experience in the development and management of stroke discharge programmes and with a certain degree of academic authority and influence were selected. The number of consulting experts is mainly affected by the research questions and available resources, which should theoretically ensure that the prediction will lead to the required evaluation results, and 15–50 experts are generally appropriate (21). According to the characteristics and actual situation of this study, 20 consulting experts were purposefully selected.

Experts who met the inclusion criteria were discussed and debated with the members of the group to form the first round of expert consultation questionnaire. The first round of the consultation questionnaire consisted of four parts. The importance of each item in the questionnaire was scored on a Likert 5 scale. A modification column was also included after each indicator item in order to know the experts’ opinion on the modification of the indicator. The study questionnaires were distributed and returned via email. The returned questionnaires were checked and sorted out in a timely manner, and if there were omissions or missing items in the questionnaires, the experts were contacted in time to complete them. The team summarised, collated and analysed the results of the first round of consultation and the modifications made by the experts, and revised and supplemented the questionnaire to form a new consultation questionnaire. The second round of consultation questionnaire of this study also included four parts: the first, second and fourth parts were the same as the first round of consultation questionnaire, and the third part was modified and deleted from the dimensions of KAP, respectively. The second round of expert consultation was also distributed and collected by email, and the experts were told to return the questionnaires within 1 week. After the two rounds of experts’ consultation, the experts’ opinions converged. According to the purpose of this study combined with the literature, the mean, coefficient of variation, and full score rate of each item were calculated according to the indicator importance scores. The mean ≥ 3.5, coefficient of variation ≤ 0.25, and full score rate ≥ 75% were used as the criteria for retaining the items in this study (22).

#### Pre-survey

A total of 60 clinical nurses were selected by convenient sampling method in 2 Level-3 hospitals in Chengdu for pre-investigation in March 2022. Inclusion criteria were as follow: ① nurses who have been working in the stroke ward for 1 year; ② aged ≥18 years; ③ able to make correct judgement on the meaning of each item in the questionnaire; ④ agreed to participate in the survey. The nurses’ KAP questionnaire for discharge planning of stroke patients was prepared by the research group. The questionnaire consisted of two parts: the first part was a general data of the survey respondents, and the second part was a questionnaire on nurses’ KAP about discharge planning for stroke patients, which was formally determined by two rounds of the Delphi expert consultation method, and consisted of a total of 37 items in the three dimensions of nurses’ KAP. The above questionnaire was distributed in the form of a questionnaire star to the nurses who fulfilled the inclusion criteria, and the same questionnaire was distributed to the 60 nurses ten days later to assess the re-test reliability of the questionnaire.

#### Reliability and validity tests

Reliability: Internal consistency was measured by using Cronbach’s alpha coefficient, which was used to analyze each dimension and observe the change of coefficients after deletion of the corresponding items.

Content validity (CVI): Five members of the expert group were invited to carry out the test of CVI again, and the CVI values of each item and all items were obtained.

### Investigating nurses’ KAP for discharge planning of stroke patients

Clinical nurses caring for stroke patients in 24 Level-3 hospitals across the country were selected from May to June 2022 to complete the questionnaire, which was distributed using a questionnaire star. Multistage sampling (also called multistage sampling) was adopted for random sampling. In this study, excluding Hong Kong, Macao, and Taiwan, the other 6 regions were sampled, and a total of 24 tertiary hospitals were sampled according to the successive multistage sampling of provinces/municipalities directly under the central government (3–4 each), cities (1 each), and hospitals (1 each). Inclusion criteria included: ① nurses who have been working in the stroke ward for 1 year; ② aged ≥18 years; ③ able to make correct judgement on the meaning of each item in the questionnaire; ④ agreed to participate in the survey. The sample size of the survey was estimated by calculating 10–15 times the total number of questionnaire items (23). The questionnaire prepared in this study had 37 items, taking 15 times the number of items, considering 10% of invalid questionnaires, the final number of surveyed people was determined to be 611, and the actual number of surveyed people was 634.

The questionnaire consisted of 37 items in three dimensions: knowledge, attitude and practice, including 12 items in the knowledge dimension, which were rated on a Likert 5 scale, with each item scored from 1 to 5 and the total score being 60, with a higher score indicating a higher degree of knowledge acquisition; and 10 items in the attitude dimension, which were also rated on a Likert 5 scale, with each item scored from 1 to 5 and the total score being 50. The higher the score, the more positive the nurse’s attitude; The 15 items in the practice dimension were also scored on a Likert 5 scale, with each item scored from 1 to 5, and the total score was 75, and the higher the score, the greater the tendency to positive and healthy practice. In this study, the dimension score = (actual score/highest score of the dimension) × 100%, and the results of the dimension scores were divided into three grades: good >85 points, moderate 60–85 points, and poor <60 points (24).

The questionnaire was distributed and recovered using the questionnaire star. After contacting the person in charge of each hospital in advance and with the consent of the head nurse of the department, the nurses were centralised to fill in the questionnaire under the unified arrangement of the head nurse. The results of the survey were monitored in the background of the mobile phone, and at the same time, it was set that each mobile phone could only answer once in case of repeated answers, and the nurses’ answer time was 1 week.

Ethical approval was granted by the institutional review board of the West China Hospital. All participants were informed about the purpose and procedure of the study and their freedom to withdraw from the study any time was assured. Written informed consent was obtained from all participants. All methods were carried out in accordance with relevant guidelines and regulations.

### Statistical analysis

All data were inputted into the Epi Data 3.0 software, and expert coordination was utilized to analyze and evaluate the reliability and validity of the data using SPSS25.0 and Lisrel 8.7 statistical software. The questionnaire’s reliability was assessed through retest reliability, split-half reliability, and Cronbach’s *α* coefficient, while the validity of the scale was evaluated through content validity, structural validity, and factor analysis. It is widely accepted that an expert with an authority coefficient greater than 0.7 is considered credible. The degree of coordination refers to how consistent experts are in their judgments on each indicator. This is often measured using Kendall’s W. The reliability of a questionnaire is reflected in its internal consistency reliability, measured by the Cronbach’s *α* coefficient. The closer the coefficient is to 1, the better the reliability. A coefficient of over 0.9 is considered very good, over 0.7 is considered good, and below 0.4 is considered poor. CVI: After forming the questionnaire, we consulted 20 experts by letter to evaluate 37 indicators. The average CVI was calculated using the phase response formula and was found to be 0.972. The software package Lisrel 8.7 should be utilized to deliver the maximum likelihood factor analysis results of scale model. Confirmatory factor analysis should then be conducted to verify whether exploratory factor analysis model is consistent with the data.

## Results

### Expert initiative

Twenty expert letter consultation forms were sent out in both rounds, and 20 valid questionnaires were collected in each round, resulting in a 100% effective recovery rate. This indicated a high level of enthusiasm among the experts. During the first round of expert letter consultation, 6 knowledge items and 6 attitude items were removed, while 10 items were modified or adjusted. The second round of expert letter consultation questionnaire consisted of 2 primary indicators (nurses’ KAP for discharge planning of stroke patients) and 37 tertiary indicators. Based on the second round of expert opinions, 2 items were removed and 3 items were modified, resulting in the formation of the “Questionnaire on Nurses’ KAP for Discharge Planning of Stroke Patients.” This questionnaire consists of 3 dimensions and 37 items, and uses a Likert 5 scale (Table 1).

**Table 1:** Nurses’ knowledge-attitude-practice questionnaire for discharge planning of stroke patients.

Dimensions	Items
Knowledge	1. Discharge planning is a part of continuity of care and an extension of inpatient care. 2. Discharge planning is initiated within 24 h of admission. 3. The contents of discharge planning include target population screening and needs assessment of discharge planning, nurse–patient cooperation to develop personalized discharge planning, implementation of in-hospital discharge planning and continuation of out-hospital discharge planning. 4. Discharge planning is a collaborative process of multidisciplinary teams. 5. The multidisciplinary team comprises doctors, specialist nurses, pharmacists, social workers, rehabilitators, psychotherapists and other health service personnel. 6. The discharge planning team includes patients and their families. 7. The discharge planning is jointly managed by the team members, and a specific coordinator is identified within the team to coordinate the discharge planning process. 8. Discharge planning documents include risk screening tools, flow charts, referrals, summaries, etc. 9. The discharge planning process includes four processes: evaluation, plan, implementation and evaluation. 10. The contents of discharge planning evaluation included patients’ physical and mental aspects, social and cultural background, family support, home environment and social resources. 11. The formulation and implementation of discharge planning is divided into two stages: inside and outside the hospital. 12. The effect of discharge planning implementation should be tracked by phone within 7 days of discharge, and again after 2 weeks or 1 month after discharge.
Attitude	1. Discharge planning reduces the rate of unplanned readmissions. 2. Discharge planning increases patient and caregiver satisfaction. 3. Discharge planning can reduce the cost of acute medical and community health services, thereby achieving population health goals. 4. Discharge planning is one of the important measures to improve patients’ life quality and promote their life safety. 5. Discharge planning plays an important role in saving social and family human resources and improving doctor-patient relationship. 6. Hospitals coordinate the use of various medical institutions, facilities and social resources to maintain the continuity of discharge planning implementation. 7. The nurse should lead the multidisciplinary team through the initial assessment and give the caregiver the support they may need. 8. The nurse provides individualized discharge planning according to the patient’s care needs. 9. Hospitals (or departments) should carry out formal training on stroke discharge planning knowledge, and guide community staff to assist supervisors in completing the plan. 10. The knowledge of stroke discharge planning is of great help to my work.
Practice	1. The need for discharge planning services is assessed within 24 h of admission. 2. Reevaluate the clients of the discharge planning within 72 h of admission. 3. Assess the patient’s physiological, psychological, socio-cultural background, family support, home environment, etc. 4. Assess whether the patient needs post-discharge services and the type, items, and frequency of services. 5. Assess the educational level and needs of patients and related personnel, and implement appropriate training and technical support. 6. Our team members work together to make the discharge planning. 7. Provide personalized quality nursing services, realize the seamless connection of patients from the hospital to the home (or community). 8. Provide discharge planning programmes that promote patient safety and access to continuous, rational, and safe care in the next environment. 9. Daily health education for patients and caregivers according to the discharge planning list. 10. Develop and arrange rehabilitation training for stroke patients. 11. Provide referral service for patients, transfer patients valuables, drugs used, medication information, related records, etc. 12. Provide psychological intervention and emotional support for patients. 13. Follow-up work for patients, carry out personalized needs nursing. 14. Monitor the implementation status of discharge planning throughout the process, adjust and modify regularly. 15. Pay attention to some knowledge and information related to the discharge planning of stroke patients, and constantly improve my service ability.

### The degree of authority of the expert

The authority coefficient of an expert was primarily determined by the expert’s academic achievements, familiarity with the investigated problem, and the basis of their judgement. In this study, the average authority coefficient of the experts was calculated to be 0.88, indicating a high level of expertise.

### Coordination coefficient of expert opinions

In the first round, Kendall’s W was 0.328, and in the second round, it increased to 0.580 (*p* < 0.05), which was a statistically significant improvement (Table 2). This showed that the consistency and coordination among experts in the two rounds were good.

**Table 2:** Expert coordination coefficient (*N* = 20).

Round	Kendall’s W	*p*
First round	0.328	<0.001
Second round	0.580	<0.001

### Reliability test of questionnaire

In this study, the pre-survey questionnaire had a total Cronbach’s *α* coefficient of 0.909 and a retest reliability of 0.924. The Cronbach’s α coefficient scores for knowledge, attitude, and practice were 0.852, 0.959, and 0.966, respectively. These results indicated that the scale was highly stable.

### Validity test of the questionnaire

The correlation coefficients were 0.635, 0.659, and 0.566, respectively, with a *p*-value of less than 0.001 (Table 3). In this study, the Kaiser-Meyer-Olkin (KMO) value was 0.966, and the Bartlett sphericity test resulted in an approximate chi-square (χ^2^) of 17621.843, with df = 594 and *p*-value of less than 0.0001.

**Table 3:** Correlation analysis of nurses’ KAP for discharge planning of stroke patients.

Item	Knowledge	Attitude
Attitude	0.635**	–
Practice	0.658**	0.566**

** *p*-value < 0.01.

### Nurses’ KAP scores on discharge planning for stroke patients

Nurses’ knowledge of discharge planning for stroke patients was 12 items, with a maximum score of 60, a minimum score of 12, and a mean score of 50.41 ± 9.00. Nurses’ attitudes towards discharge planning for stroke patients had 10 items, with a maximum score of 50 and a minimum score of 10, and a mean score of 45.30 ± 6.43. Nurses’ practice on discharge planning for stroke patients were 15 items, with a maximum score of 75 and a minimum score of 15, with a mean score of 61.37 ± 13.27. The results of this study were calculated on a dimensional basis, resulting in the following standard scores: 84.01 (<85) for the knowledge dimension, 90.61 (>85) for the attitude dimension, and 81.83 (<85) for the practice dimension (Table 4). To determine the optimal structure of the measurement dimensions of the scale, a confirmatory factor analysis was performed on 626 samples with 37 items. The fitting reference numbers of meter modulus are as follows: *χ^2^* = 2619.99, *χ^2^/*df = 4.68, RMSEA (root mean square of approximate error) = 0.092, CFI (comparative fit index) = 0.98, GFI (goodness of fit index) = 0.77, AGFI (adjusted goodness of fit index) = 0.74, NFI (normative fit index) = 0.95, IFI (incremental fitting index) = 0.96. Additionally, the correlation coefficient between each factor was greater than 0.5.

**Table 4:** Nurses’ total KAP scores on discharge planning for stroke patients (*N* = 626).

Item	Mean ± standard deviation	Standard score (points)
Knowledge	50.41 ± 9.00	84.01
Attitude	45.30 ± 6.43	90.61
Practice	61.37 ± 13.27	81.83

## Discussion

### Reliability and validity analysis

The reliability test was helpful in understanding the reliability and validity of the questionnaire. The evaluation indexes included test–retest reliability, internal consistency reliability and split-half reliability. The reliability analysis of the pre-survey results showed that the total Cronbach’s *α* coefficient for test–retest reliability was 0.909, while the Cronbach’s *α* coefficient of the foreign Discharge Planning scale (2002) (25), which only measured the knowledge part, was only 0.7. The high internal consistency (Cronbach’s α = 0.909) surpasses the threshold of 0.9, indicating excellent reliability. This exceeds values reported in similar tools, such as the Discharge Planning Scale (α = 0.70–0.85) developed by Naylor et al. (26) for cardiac patients, underscoring the robustness of our multidimensional KAP structure. The validity test was helpful in understanding the validity and accuracy of the instrument. The evaluation indexes included content validity, structure validity, surface validity and criterion-related validity. The questionnaire indexes were constructed based on previous research, literature and two rounds of expert correspondence. The experts showed high enthusiasm, authority and coordination, resulting in a content validity of 0.972, indicating good content validity was. The CVI (0.972) and KMO (0.966) values significantly exceed conventional psychometric thresholds. This positions our tool among the most rigorously validated instruments internationally, as demonstrated in large-scale studies of discharge planning competency (13). The correlation coefficient between the three first-order indexes and the total score of the questionnaire was greater than 0.635, while the correlation coefficient between the three first-order indexes was greater than 0.5.

### The structural characteristics of the evaluation indexes of nurses’ KAP for discharge planning

According to the KAP constitution, it covers the requirements for first-line clinical nurses in the discharge planning of stroke patients. This includes basic knowledge, relevant knowledge, psychological cognition, emotional tendency, intention behavior, evaluation, implementation and evaluation of discharge planning. In addition, the demands of first-line nurses to carry out the overall, seamless responsibility system for the patients were emphasized in promoting high-quality nursing service projects. This includes providing discharge planning from admission assessment, basic care, specialist care, psychological care, health education, discharge follow-up, nurse–patient communication, and a questionnaire with 37 items in 3 dimensions of discharge planning knowledge, attitude and practice was formed. By using 37 items to evaluate the KAP of nurses in third-grade hospitals, the need for framing questions was eliminated, resulting in significantly improved authenticity and validity of survey data. The questionnaire’s structural validity was tested using the factor analysis method. The KMO value was 0.966, and the spherical test value *p* < 0.0001, indicating good structural validity. Confirmatory factor analysis was utilized to test the relationship between a set of measurement variables (observations or items) and a set of factor concepts that can explain the measurement variables (factors or measurement concepts) based on a specific theoretical or conceptual framework. Mathematical procedures were then used to confirm and evaluate whether the measurement model (factor structure) derived from the theoretical viewpoint was appropriate and reasonable. The main focus of the study was to test the applicability and authenticity of the constructional validity (27). Confirmatory analysis was employed to test the model, ensuring the rationality of the scale structure and the certainty, stability and reliability of the measured content. The fit index and correlation of each factor of the scale meet the measurement standards, indicating that the questionnaire factor model had a good match with the data and exhibited good structural validity.

### Construct the significance of nurses’ KAP questionnaire for discharge planning of stroke patients

The KAP model is an important theory in promoting human health related behavior (28). It recognizes that the final change in human behavior occurs through three successive steps: acquiring knowledge, generating belief and forming behavior. As a major player in the implementation of discharge planning for stroke patients, nurses’ knowledge, attitude and behavioral abilities directly affect the quality, safety, efficiency and satisfaction of nursing care. After discharge, stroke patients have high nursing needs, and domestic discharge planning for stroke patients is still in the initial stage of exploration. 14.3% of nurses believed the discharge planning was just paper work (25), and 40% of nurses reported that they lacked knowledge of discharge planning (29). Most domestic scholars have only investigated nurses’ cognition of discharge planning, but have not mentioned theirs’ attitude and behavior. According to the study, more than 42.9% of clinical nurses are not familiar with discharge plan and only partially familiar with discharge planning (30). This study showed that nurses’ knowledge and practice regarding discharge planning for stroke patients were at a moderate level and their attitudes were good. Overall, the level of nurses’ KAP regarding discharge planning for stroke patients needs to be improved. Therefore, this study focused on domestic clinical nurses as the research object, and constructed an evaluation tool of KAP for discharge planning of stroke patients. This not only enriched the application of KAP model in overall nursing of responsibility systems, but also helped managers understand the current situation of KAP in discharge planning of clinical nurses. As a result, targeted training can be conducted to strengthen clinical nurses’ knowledge of discharge planning, change their attitude and beliefs and ultimately achieve the purpose of behavioral change. It provided a reference for appointing and assessing discharge planning nurses, outlined their job responsibilities, certification qualifications and quality control standards. Ultimately, this led to an improvement in the quality of discharge planning work.

Several limitations of this study need to be addressed. First, we did not use cognitive testing to select items, although the use of expert panel review and testing of several items with similar meaning to select optimized items would be expected to decrease the impairment of this limitation. Second, this KAP questionnaire was somewhat geographically specific and may not be applicable to other countries or healthcare units. Therefore, future studies should: (i) validate the tool in diverse healthcare tiers (e.g., community hospitals) and cultural contexts; (ii) employ longitudinal designs to assess KAP changes post-intervention; and (iii) develop multilingual adaptations (e.g., English, Spanish) for global benchmarking using cross-cultural validation frameworks like those proposed by Sousa and Rojjanasrirat (31).

## Conclusion

In conclusion, this study developed and validated a good reliability of the nurses’ KAP questionnaire for discharge planning for stroke patients. Nurses in Level-3 hospitals across the country need to improve their knowledge, attitude and practice regarding discharge planning for stroke patients. Clinical nurses can use this KAP scale to improve their KAP of discharge planning for stroke patients, so as to better achieve the continuity of health services.

## Data Availability

The original contributions presented in the study are included in the article/supplementary material, further inquiries can be directed to the corresponding author.
